# Identification of galectin-7 as a potential biomarker for esophageal squamous cell carcinoma by proteomic analysis

**DOI:** 10.1186/1471-2407-10-290

**Published:** 2010-06-15

**Authors:** Xi Zhu, Ming Ding, Mei-Lan Yu, Ming-Xiang Feng, Li-Jie Tan, Fu-Kun Zhao

**Affiliations:** 1 Institute of Biochemistry and Cell Biology, Shanghai Institutes for Biological Sciences, Chinese Academy of Sciences, 320 Yue-Yang Road, Shanghai 200031, China; 2 Department of Thoracic Surgery, Zhong Shan Hospital, Fu Dan University, Shanghai, 200032, China; 3 College of Life Science, Zhejiang Sci-Tech University, Hangzhou 310018, China

## Abstract

**Background:**

Esophageal squamous cell carcinoma (ESCC) is one of the most common malignancies. Early diagnosis is critical for guiding the therapeutic management of ESCC. It is imperative to find more effective biomarkers of ESCC.

**Methods:**

To identify novel biomarkers for esophageal squamous cell carcinoma (ESCC), specimens from 10 patients with ESCC were subjected to a comparative proteomic analysis. The proteomic patterns of ESCC samples and normal esophageal epithelial tissues (NEETs) were compared using two-dimensional gel electrophoresis. And differentially expressed proteins were identified using MALDI-TOF-MS/MS. For further identification of protein in selected spot, western blotting and immunohistochemistry were employed.

**Results:**

Twelve proteins were up-regulated and fifteen proteins were down-regulated in the ESCC samples compared with the NEET samples. Up-regulation of galectin-7 was further confirmed by western blotting and immunohistochemistry. Furthermore, immunohistochemical staining of galectin-7 was performed on a tissue microarray containing ESCC samples (n = 50) and NEET samples (n = 10). The expression levels of galectin-7 were markedly higher in the ESCC samples than in the NEET samples (*P *= 0.012). In addition, tissue microarray analysis also showed that the expression level of galectin-7 was related to the differentiation of ESCC.

**Conclusions:**

The present proteomics analysis revealed that galectin-7 was highly expressed in ESCC tissues. The alteration in the expression of galectin-7 was confirmed using a tissue microarray. These findings suggest that galectin-7 could be used as a potential biomarker for ESCC.

## Background

Esophageal carcinoma is one of the most malignant gastrointestinal cancers and is ranked the 6th leading cause of cancer death worldwide [1]. Esophageal squamous cell carcinoma (ESCC) is the predominant histological subtype of esophageal carcinoma in Asia [2]. The overall 5-year survival rate of ESCC is less than 10% [3]. One of the primary reasons for this high mortality rate is that ESCC is often not detected until it has invaded surrounding organs and is therefore at an advanced stage. Surgery is inappropriate in 40-60% of patients due to unresectable disease status, the presence of distant metastases, or high operative risk [4]. Conventional chemotherapy and radiotherapy treatments are also relatively ineffective [5]. The odds of long-term survival are poor when the disease extends through the esophageal wall or when it is diagnosed with widespread lymph node involvement. Early diagnosis and exact histological grading of ESCC are therefore critical for guiding the therapeutic management of ESCC.

A biomarker is a molecule that indicates a pathological alteration in physiology. Cancer biomarkers can provide important information for cancer diagnosis and staging and give guidance in many areas, such as cancer therapy [6]. High-throughput and high-sensitivity proteomics technology provides an effective approach for screening novel cancer-specific biomarkers. Tissue-based proteomic analyses directly relate protein biomarkers to disease and have been widely used in the study of various tumors [7-9]. In past years, molecules identified by proteomics, such as pRB protein, tropomyosin isoform 4 (TPM4), prohibitin and periplakin [10-14] have been reported as potential biomarkers for the diagnosis of ESCC. However, most of these molecules require experimental verification using clinical-scale sample sets before clinical application. Thus, it is still imperative to find more effective, clinically verified biomarkers of ESCC [15].

Galectins are a family of β-galactoside-binding lectins with diverse biological functions. Different galectin family members have been reported to be show significantly altered expression [16,17] and play important roles in various types of cancer [18,19]. In a recent report, induction of galectin-1 expression by the human pituitary tumor transforming gene (PTTG) promoted lymph node metastasis in human ESCC [20]. Galectin-7, a member of the galectin family initially identified in human epidermis, is a 15-kDa protein with a single carbohydrate recognition domain [21,22]. Expression of galectin-7 is restricted to stratified epithelial cells including the skin, tongue, esophagus and Hassal's corpuscles in the thymus [23]. The major functions of galectin-7 include regulation of cell-cell and cell-matrix interactions, apoptosis and immunity [16,24,25]. Growing evidence indicates that galectin-7 plays an important role in cancer progression [26-28]. Galectin-7 has been reported to function as an important molecule in the dissemination and invasion of lymphoma cells in human lymphoid malignancies [29]. It is also involved in muscle infiltration of urothelial cancer [30]. However, the expression pattern of galectin-7 in ESCC tissue is still unknown.

In this study, a proteomics approach was used to analyze the different proteomic patterns in ESCC and NEET samples. After comparing the expression patterns, differentially expressed proteins between them were identified. Among these proteins, galectin-7 was found to be highly expressed in ESCC tissues. Confirmatory studies have been done to examine the possibility of galectin-7 to be a biomarker for ESCC.

## Methods

### Materials

The ESCC specimens were obtained from 10 patients who undergone surgery in the thoracic surgery department of Zhongshan Hospital. All ESCC patients undergone curative resection and were not treated with neoadjuvant chemotherapy or radiotherapy. The clinical staging was performed according to the tumor-node-metastasis classification system. The overall clinicopathological data of the cases are listed in Table 1. Matched normal mucosal tissues located at least 5 cm away from the tumor margins were also included in this study. Two additional groups of ESCC cases were used in this work, 16 cases for immunohistochemistry, 50 cases for tissue microarray, which included 12 cases categorized as highly differentiated, 14 cases categorized as moderately differentiated, 13 cases categorized as poorly differentiated, and other cases in which pathologists did not reach a consensus as to the degree of differentiation. Freely tendered informed consent was obtained from all patients. The study was approved by the local ethics committee of Zhongshan Hospital.

**Table 1:** Clinicopathological data of the 10 ESCC cases

Sample No.	Age	Gender	Site	Pathological grade	Size (cm)	Lymphatic invasion	TNM stage
1	58	Male	Upper	Moderately differentiated	3	Negative	II
2	67	Female	Middle	Moderately differentiated	5	Positive	III
3	55	Male	Middle	Well differentiated	2	Negative	I
4	62	Male	Middle	Moderately differentiated	2	Negative	I
5	62	Male	Upper	Moderately differentiated	2.5	Negative	II
6	52	Male	Middle	Poorly differentiated	4	Negative	II
7	61	Male	Middle	Moderately differentiated	3	Negative	II
8	55	Female	Lower	Poorly differentiated	6	Positive	IV
9	70	Male	Lower	Moderately differentiated	5	Positive	III
10	53	Male	Middle	Poorly differentiated	3	Positive	II

The IEF system (IPGphor), ImmobilineDryStrips (24 cm, pH 3-10NL) and CLEAN-UP Kits were purchased from Amersham Biosciences. Iodoacetamide was obtained from Sigma-Aldrich (St. Louis, MO). The MS experiments were carried out using an ABI 4700 MALDI-TOF/TOF mass spectrometer (Applied Biosystems, Framingham, MA). Sequence-grade trypsin was obtained from Promega (Madison, WI, USA), and the standard peptide mixture used for calibration was obtained from Applied Biosystems. All buffers were prepared with Milli-Q water.

### Sample preparation

Each tissue sample was crushed and ground in a mortar containing liquid nitrogen. The resulting powder was immediately suspended in a lysis buffer (7 M urea, 2 M thiourea, 4% CHAPS, 65 mM DTT, 2% IPG buffer, and trace cocktail protease inhibitor). After the solution was vigorously stirred for 1 hr, the cell debris and insoluble substances were removed by centrifugation at 100,000 g for 45 min. Protein concentrations were determined using a modified Bradford method [31]. The supernatant was aliquoted and stored at -80°C for further use.

### Two-dimensional electrophoresis (2-DE)

Seven batches of tissue samples were subjected to 2-DE. 2-DE was carried out according to the manufacturer's instructions [32] with minor modifications. Briefly, 200 μg of protein sample was diluted to 450 μL with a rehydration solution (7 M urea, 2 M thiourea, 0.2% DTT, 0.5% [v/v] pH 3-10 IPG buffer and a trace of bromphenol blue) and applied to IPG strips (24 cm, pH 3-10NL) in a 14-h rehydration. Proteins were focused on an IPGphor IEF system (Amersham Biosciences) for a total of 61 kVh. The two-dimensional separation was performed on 12.5% polyacrylamide gels with an Ettan DALT twelve apparatus (Amersham Biosciences) at 25°C. Protein spots in the gels were visualized by silver staining as described by Yan *et al *[33] with minor modifications.

### Image analysis

Silver-stained 2-DE gels were scanned using a D2000 Uniscan scanner (Tsinghua Uniscan, Beijing, China) with 300-dpi resolution and analyzed using ImageMaster™ 2D Platinum software (Version 5.0; Amersham Bioscience). To guarantee the reliability of the results, the gel images of seven batches of samples were imported to ImageMaster™ 2D Platinum software simultaneously. Protein spots were automatically detected. Individual spot volumes were normalized against total spot volumes. Alignment and matching of the spots were carried out by choosing one gel as a reference and by manually selecting one common spot as a landmark. Two-sample t-tests were used to analyze differences in protein expression between the ESCC and NEET groups. Fold changes more than 1.5 and P values less than 0.05 were considered statistically significant.

### In-gel digestion and MS

The selected protein spots were excised from the gels and incubated in a silver-destaining solution containing 30 mM potassium ferricyanide and 100 mM sodium thiosulfate (1:1) for 10 min at room temperature. The samples were washed twice with Milli-Q water and once with 25 mM ammonium bicarbonate/50% acetonitrile (ACN). The samples were then dehydrated in 100% (v/v) ACN and dried in a speed-vacuum. After drying, the samples were rehydrated on ice for 45 min in 2.5 μl of 25 mM NH_4_HCO_3 _containing 10 ng/l sequencing-grade trypsin (Promega, Madison, WI). The rehydrated gel spots were digested for 4 hrs at 37°C. The peptide fragments were extracted with 5% TFA and 2.5% TFA/50% ACN in succession. The supernatants were pooled, dried, and recovered in 1.5 μL of 0.5% TFA. Finally, the digested peptide samples were co-crystallized with an equal volume of saturated matrix solution (CHCA in 0.1% TFA in H_2_O/ACN [2:1]) on the MALDI sample target plate. Peptide mass spectra were obtained with a MALDI-TOF/TOF mass spectrometer (4700 Proteomics Analyzer, Applied Biosystems).

The protein spots were identified by MS and MS/MS as described by Tian *et al *[34]. Prior to real sample acquisition, six external standards (mass standard kit for the 4,700 proteomics analyzer calibration mixture, Applied Biosystems) were used to calibrate each spectrum to a mass accuracy of within 5 ppm for the MS Reflector Positive Operating Mode or within 10 ppm for the MS-MS 1 KV Positive Operating Mode. A combined database search of MS and MS/MS measurements was performed using GPS Explorer™ software (Version 3.5; Applied Biosystems) and MASCOT software (Version 2.0; Matrix Science, London, UK). Searches were performed with carbamidomethylation of cysteine and oxidation of methionine as variable modifications. One trypsin miscleavage was allowed. The peptide mass tolerance and fragment mass tolerance were set to 50 ppm and ± 0.1 Da, respectively. Peptide mixtures that yielded statistically significant search scores (> 95% C.I., equivalent to MASCOT expected value < 0.05) and accounted for the majority of ions present in the mass spectra were defined as positive identifications.

### Western blotting

For validation experiments, a new set of tissue samples was collected for immunoblotting analysis of galectin-7. All protein samples for validation were separated by SDS-PAGE with 20 μg protein per lane and transferred onto a PVDF membrane (Millipore) in transfer buffer (pH 11.0, 25 mM Tris, 0.2 M glycine, 20% [v/v] methanol) for 45 min at 1.5 mA/cm^2 ^on a semidry electroblotter (Bio-Rad). After being blocked with 1% skim milk for 1 hr at 25°C, galectin-7 was detected by incubating the samples with a goat polyclonal anti-galectin-7 antibody at a 1:300 dilution (R&D Systems, Minneapolis, MN) overnight at 4°C. Horseradish peroxidase-conjugated rabbit anti goat Ig at a 1:3,000 dilution (AMS Biotechnology, Oxon, UK) was used as a secondary antibody. The immunoblots were developed using ECL detection reagent (Pierce Chemical).

### Immunohistochemistry

Immunohistochemistry was performed on formalin-fixed, paraffin-embedded tissue sections using a standard immunohistochemical technique. Four-micrometer-thick tissue sections were deparaffinized in xylene, rehydrated in a graded ethanol series and treated with an antigen retrieval solution (10 mM sodium citrate buffer; pH 6.0). The sections were incubated with a goat polyclonal anti-galectin-7 antibody (dilution 1:125) overnight at 4°C and then incubated with a 1:1,000 dilution of biotinylated secondary antibody, followed by avidin-biotin peroxidase complex (DAKO), according to the manufacturer's instructions. Finally, tissue sections were incubated with 3,3'-diaminobenzidine (Sigma-Aldrich) until a brown color developed, and the sections were then counterstained with Harris modified hematoxylin. For the negative controls, the primary antibodies were omitted.

### Tissue microarray

The tissue microarray slides (Shanghai Biochip Company, Ltd., Shanghai, China) contained 120 formalin-fixed, paraffin-embedded tissue samples, including 50 ESCC tissues, 10 NEET samples. Each sample had two sample dots. Immunohistochemistry for galectin-7 was performed using the avidin-biotin complex method (ABC; Vector Laboratories, Burlingame, CA), including heat-induced antigen-retrieval procedures. Incubation with polyclonal antibodies against galectin-7 (1:100 dilution; R&D Systems, Minneapolis, MN) was done at 48°C for 18 hours. Fourteen negative controls were treated identically but with the primary antibody omitted.

### Evaluation of galectin-7 expression

The German immunohistochemical scoring (GIS) system was applied to estimate galectin-7 expression. The GIS system is a semiquantitative scoring system in which the final immunoreaction score is expressed as the product of the intensity and quantity scores. The extent percentage of positive cells was graded as follows: 0, negative; 1, < 10% positive cells; 2, 11% to 50% positive cells; 3, 51% to 80% positive cells; 4, > 80% positive cells. The staining intensity was graded as follows: 0, negative; 1, weakly positive; 2, moderately positive; and 3, strongly positive. GIS score ≥ 6 was defined as high expression. GIS score < 6 was defined as low expression. All staining was independently evaluated by three different pathologists who were blinded to patient outcomes. Statistical analysis of GIS scores was done using SigmaPlot software. *P *values less than 0.05 were considered statistically significant.

## Results

### 2-DE proteome maps of NEET and ESCC specimens

**Figure 1:**
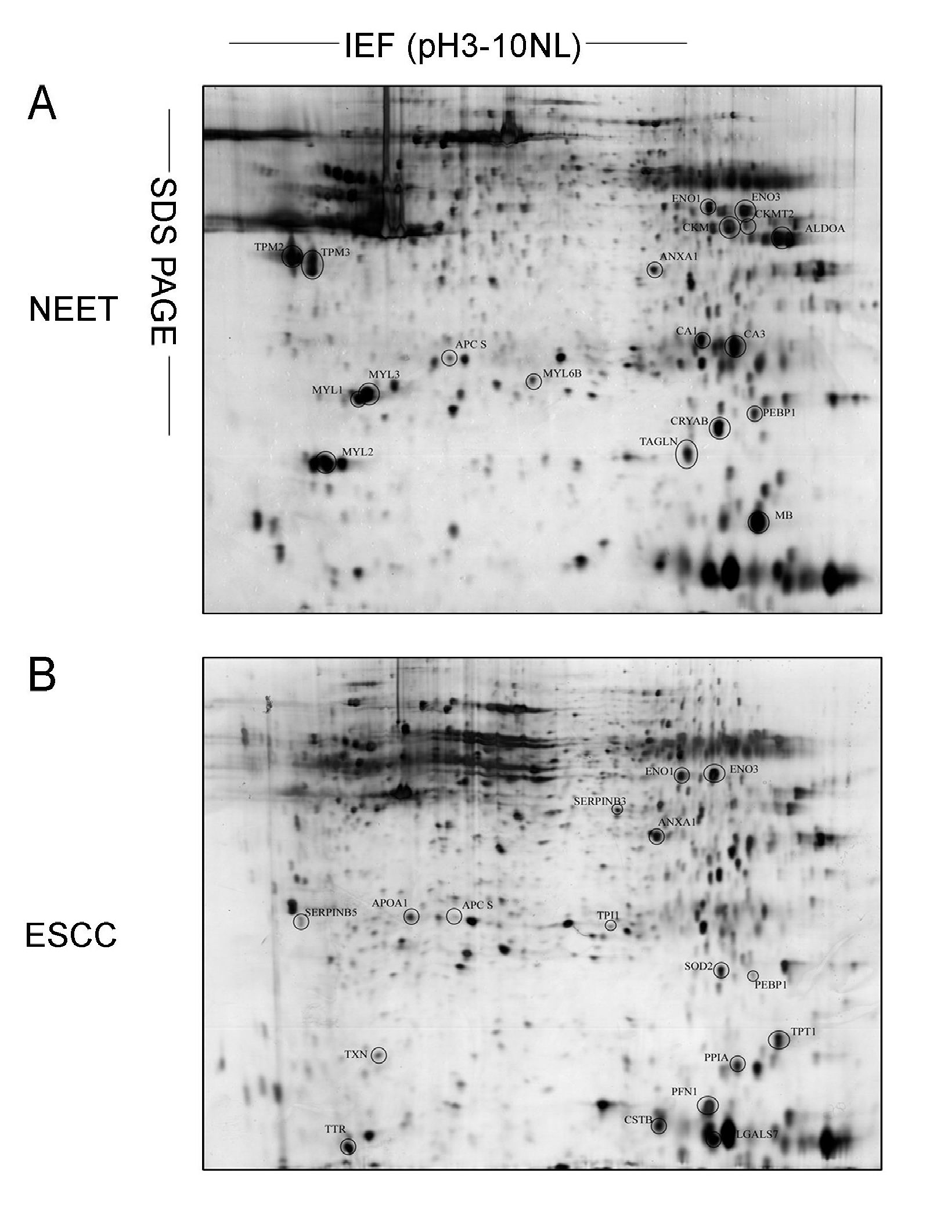
**Representative 2-DE protein profiles**. 2-DE protein profiles of NEET (A) and ESCC samples (B). Proteins were separated on the basis of pI (X-axis) and molecular mass (Y-axis). A total of 250 μg protein was separated using 24 cm pH 3-10NL IPG strips (cup-loading method) and 12.5% SDS-PAGE. Proteins were visualized by silver staining. Differentially expressed proteins are indicated with black circles. These proteins were identified by MALDI-TOF/TOF MS, as summarized in Additional file 1.

**Figure 2:**
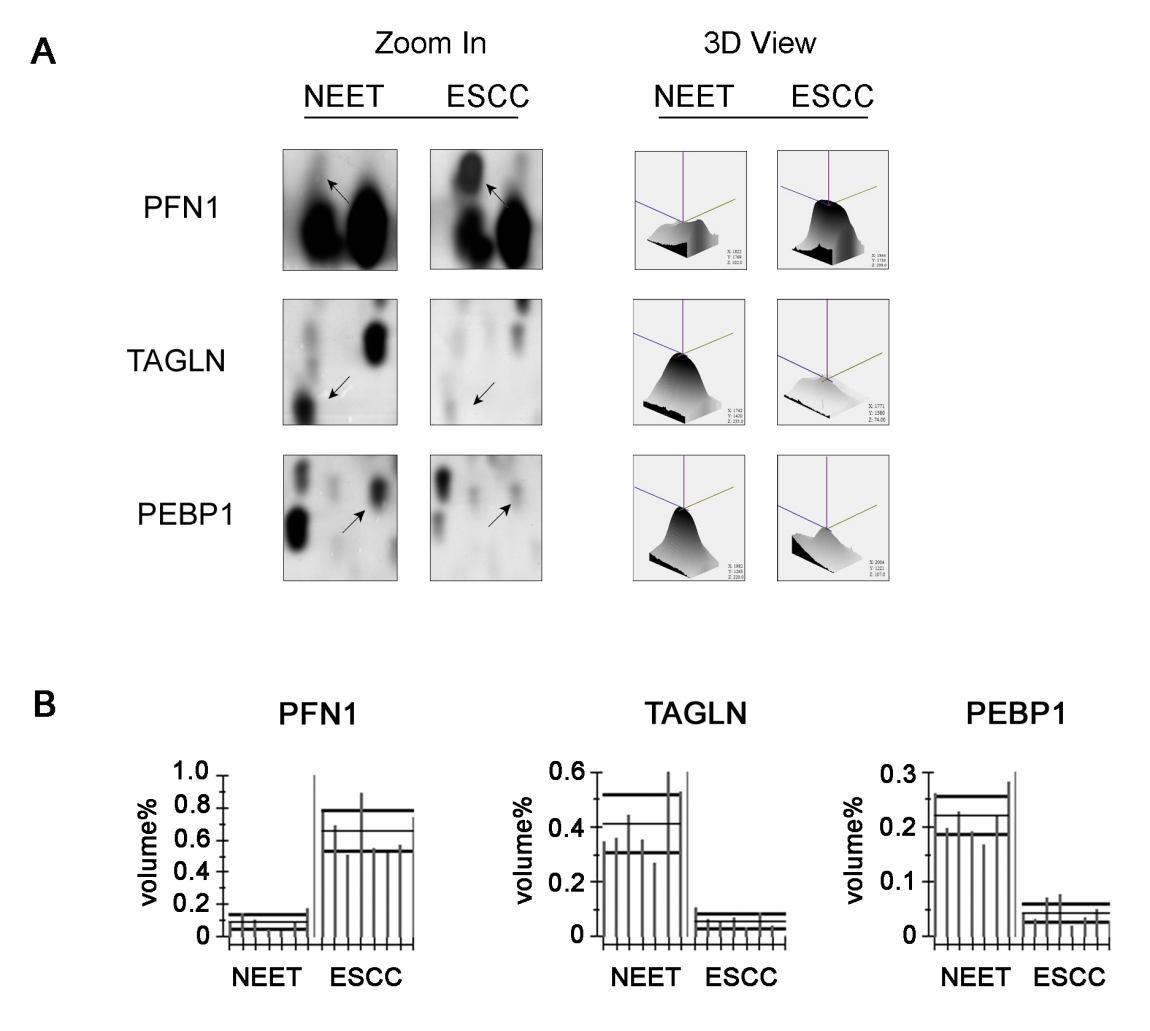
**Comparative analysis of representative proteins with altered expression patterns in NEET and ESCC cells**. (A) Magnified comparison maps and quantified 3D views. (B) Statistical analyses of the protein spots.

The proteomic profiles of the ESCC and NEET samples were examined using 2-DE. After silver nitrate staining, 2-DE images with high resolution and reproducibility were obtained. Representative 2-DE images are shown in Figure 1. To ensure reliability and reproducibility, seven batches of tissue samples were subjected to proteomic analysis using ImageMaster 2D Platinum Version 5.0 software and fourteen gels were generated. Twenty-seven protein spots had significantly different expression patterns between the two groups (> 1.5-fold change, *P *< 0.05; Figure 1). Relative to the NEET group, the expression levels of 12 protein spots were markedly increased and 15 protein spots were decreased in the ESCC group. The zoom-in and 3D quantified views showed some representative differentially expressed protein spots between the two types of tissues (Figure 2A). The statistical analysis of the volumes (% volume) of these spots from seven batches of tissue samples is shown in Figure 2B.

### Identification of differentially expressed proteins using MALDI-TOF/TOF mass spectrometry and functional exploration of candidate proteins

**Figure 3:**
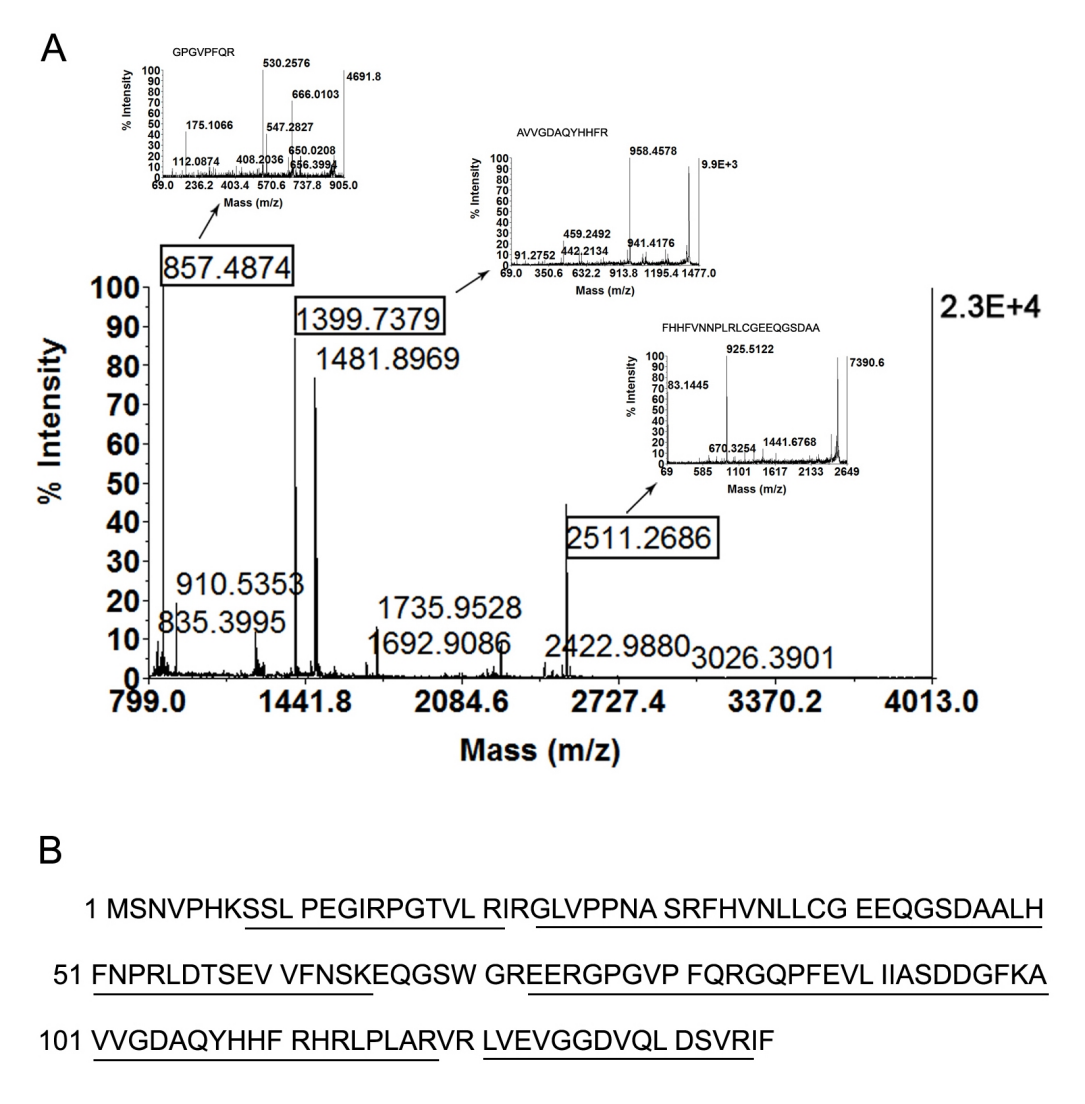
**Identification of galectin-7 by MALDI-TOF/TOF mass spectrometry**. (A) Peptide mass fingerprints of galectin-7 are shown. Matched peptide peaks are labeled with mass values, and the sequences of precursor peptides with m/z 857.4628, 1399.7379 and 2511.1833 were confirmed using MALDI-TOF MS/MS. (B) Amino acid sequences of galectin-7. Matched peptides are underlined.

To characterize the differentially expressed proteins, the 2-DE spots were excised from the silver-stained gels and analyzed by MALDI-TOF mass spectrometry. The peptide mass fingerprints (PMF) of the differentially expressed proteins were acquired in the MS Reflector Positive Operating Mode and then subjected to SwissProt database searching using GPS Explorer™ software. To further confirm the MS identification results, four peaks with the strongest signals in the MS spectra were automatically selected and subjected to MS/MS analysis. MS and MS/MS identification of each differentially expressed protein from different batches of 2-DE gels were repeated at least three times. As a representative example, the PMF and MS/MS spectra of galectin-7 are illustrated in Figure 3. The matched amino acid sequences of galectin-7 are underlined. Three precursor peptides of m/z 857.4628, 1399.7379 and 2511.1833 were confirmed as the tryptic peptides of galectin-7. The amino acid sequences of these peptides identified by MS/MS were "^76^GPGVPFQR^83^", "^100^AVVGDAQYHHFR^111^", and "^33^FHVNLLCGEEQGSDAALHFNPR^54^". Additional file 1 (Identification of proteins differentially expressed in esophageal cancer specimens by MALDI-TOF MS and MS/MS) summarizes the identified proteins and their gene names, Swiss-Prot accession numbers, theoretical molecular weights/p*I *values, mascot scores, peptide counts, coverage (%), sequence confirmation by MS/MS and MS/MS scores.

The expression patterns of differentially expressed proteins in esophageal cancer specimens and their biological functions are shown in additional file 2: Expression patterns of differentially expressed proteins in esophageal cancer specimens and their biological functions. Regulatory factors and statistics are also included in additional file 2. The identified proteins were classified into 8 functional categories based on their molecular functions and biological processes according to the KEGG and ExPasy http://www.expasy.org/sprot/ databases (See additional file 3: Functional classification of proteins). Approximately 27% of the proteins are involved in metabolism, including energy, carbohydrate, nucleotide and lipid metabolism. Cell growth and death (20%) and cytoskeletal (19%) functions accounted for the second and third categories of proteins, respectively. Proteins involved in signal transduction (11%), folding (9%), transcription (8%) and transport (2%) were also identified.

### Validation by western blotting and immunohistochemistry

**Figure 4:**
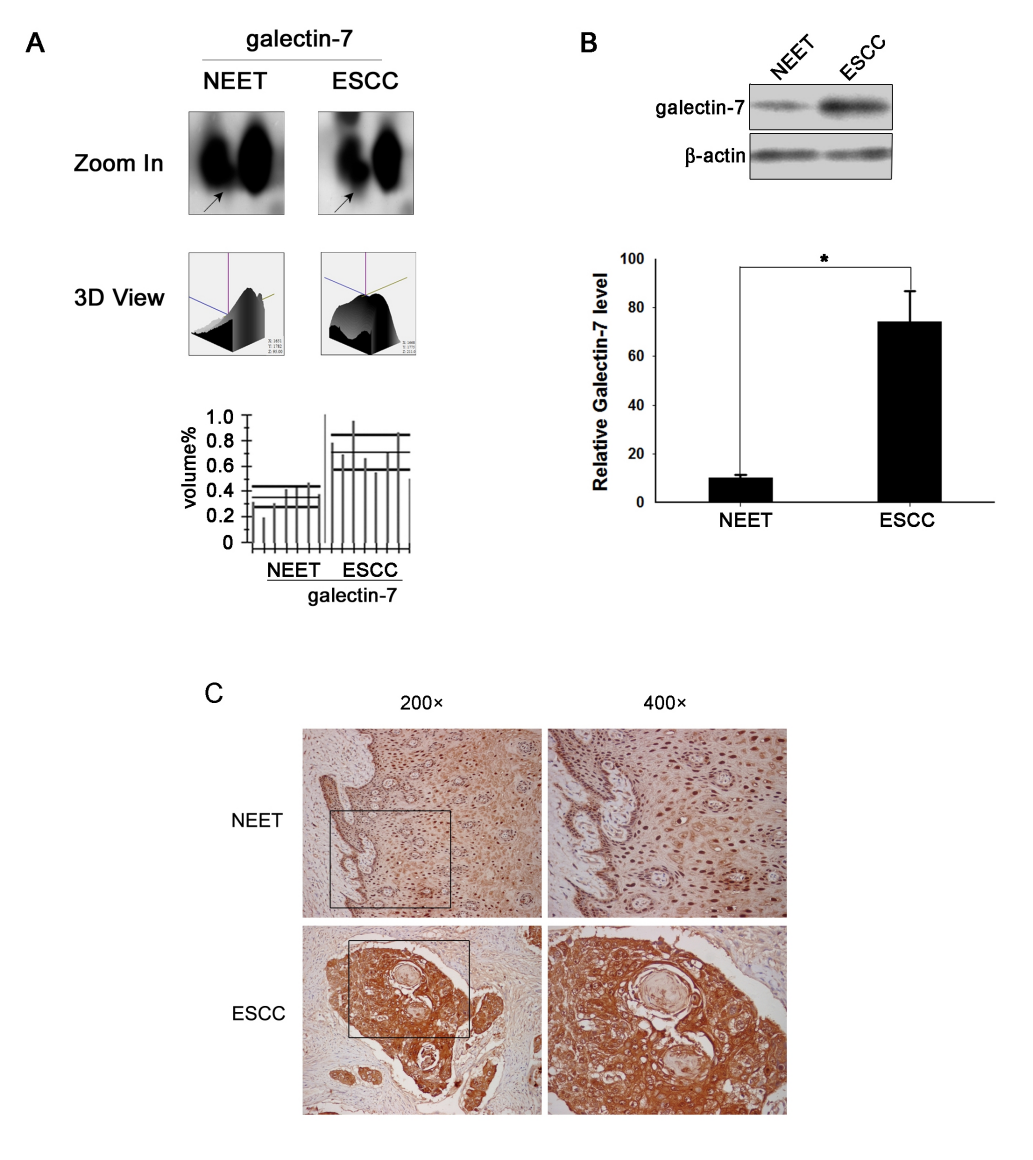
**Expression of galectin-7 is up-regulated in ESCC tissues relative to NEET tissues**. (A) The magnified comparison maps, quantified 3D views and statistical analyses of galectin-7 expression in NEET and ESCC samples. (B) Representative samples of ESCC and NEET were resolved on 10% polyacrylamide gels and immunoblotted with an anti-galectin-7 antibody and a β-actin antibody as a loading control. The intensity of each band was measured with GraphPad Prism 4.0 software, and relative galectin-7 protein levels between ESCC and NEET samples were normalized against those of β-actin. The expression of galectin-7 was increased in tumor tissues. The difference in galectin-7 expression between ESCC and NEET samples was assessed using Student's *t*-test (*P < 0.05) for unpaired values. (C) Immunohistochemical analysis of galectin-7 expression in ESCC. Galectin-7 displayed positive staining in ESCC samples that was much stronger than the staining in non-neoplastic tissue. In normal esophageal epithelial tissues, galectin-7 is localized primarily to the nucleus. Expression of galectin-7 was detected ubiquitously in the cytoplasm, nuclei and membranes of ESCC cells. Images in the left panel were taken under 200× magnification; the right panel images are 400× magnifications of the same sections depicted on the left.

The proteins identified by mass spectrometry such as annexin A1, tropomyosin, profiling 1, translationally controlled tumor protein and serpin B3 have been previously reported to be altered in malignancies [14,35-39]. However, the expression level of most of them have not been studied in ESCC. Among the differentially expressed proteins in ESCC, we decided to focus on further confirming the expression patterns of Glectin-7, whose expression levels have not been well studied in human esophageal cancers and whose change in its expression could potentially be served as a marker for ESCC. To confirm the differential expression pattern of galectin-7, additional samples were examined by western blotting and immunohistochemistry. Consistent with the 2-DE data, the western blotting results (See additional file 4: Western blotting results of galectin-7 expression in several pairs of ESCC and NEET samples) showed elevated expression levels of galectin-7 in the ESCC samples relative to the NEET samples (Figure 4B).

Immunohistochemical staining also showed that galectin-7 is highly expressed in ESCC tissues relative to NEET samples. Furthermore, the expression of galectin-7 was distributed in the cytoplasm, nuclei and membranes of ESCC cells, whereas it was distributed primarily in only the nuclei of NEET cells (Figure 4C). The sub-cellular localization and expression of galectin-7 were examined by immunofluorescence staining in four ESCC cell lines as well (See additional file 5: Sub-cellular localization of galectin-7 in four ESCC cell lines). These results, taken together with the proteomics observations, suggest that galectin-7 is up-regulated in ESCC tissues, which further support that galectin-7 could be served as a potential marker for ESCC.

### Tissue microarray

**Figure 5:**
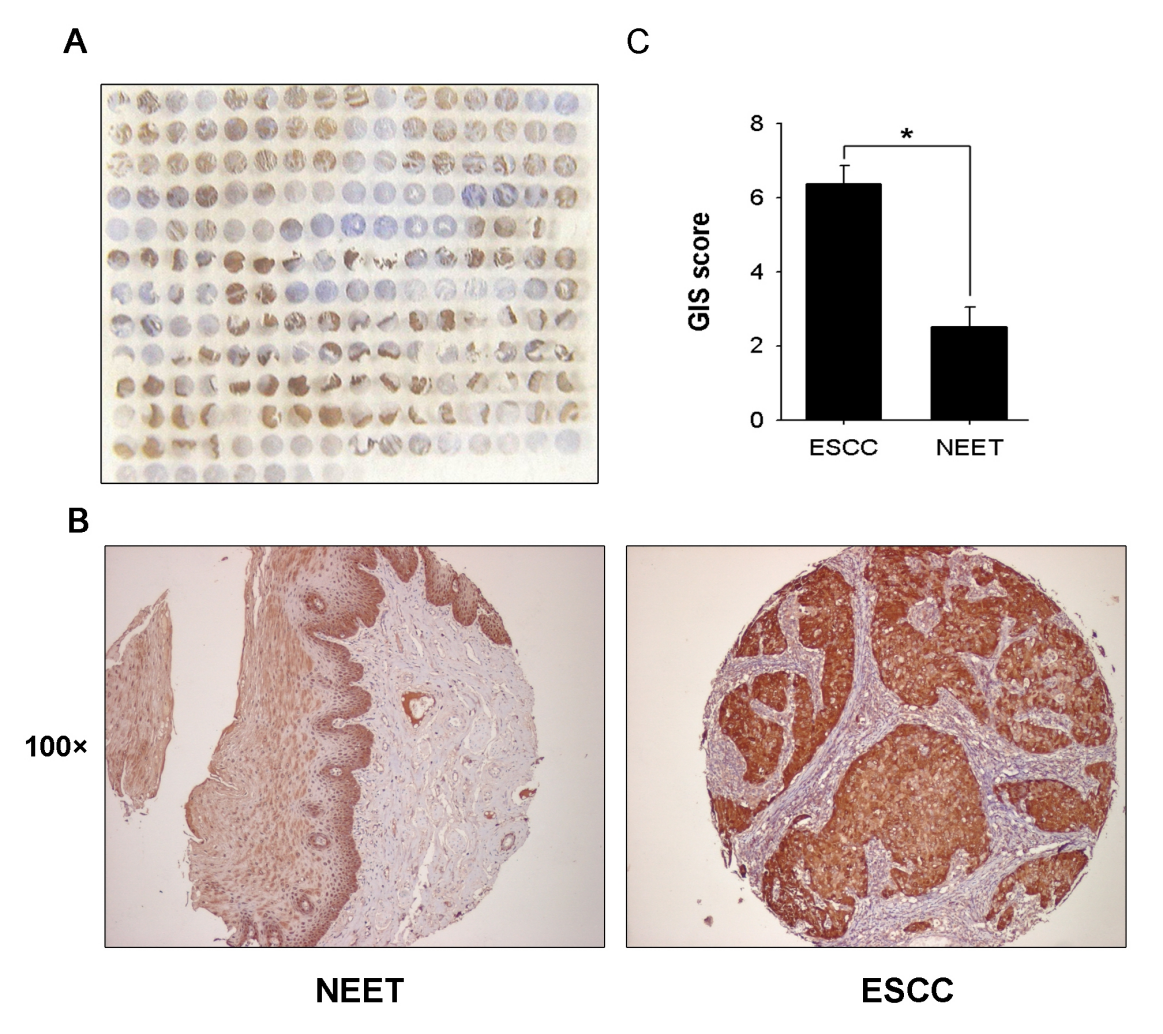
**Tissue microarray analysis of galectin-7 expression**. (A) Overview of the tissue microarray. (B) Representative example of galectin-7 expression in NEET (100×) and in ESCC tissues (100×). (C) Expression patterns of galectin-7 in NEET and ESCC were analyzed using the GIS score system. The differences in galectin-7 expression levels in ESCC and NEET were assessed by the Student's *t*-test (*P < 0.05) for unpaired values.

To further investigate the clinical significance of the differential expression of galectin-7 in ESCC tissues, we extended our studies by performing tissue microarray evaluations on a larger patient population. Figure 5 displays representative images of immunostaining for galectin-7. In 10 NEET samples, negative (n = 2, 20%), very weak (n = 7, 70%) or weak (n = 1, 10%) expression of galectin-7 was detected. In contrast, high expression of galectin-7 was detected in 28 of 50 (56.0%) ESCC samples. The fold difference of galectin-7 expression in ESCC versus NEET samples was 2.5 (*P *= 0.012) (Figure 5).

In 50 cases of ESCC, 12 cases were diagnosed as being well differentiated, 14 were diagnosed as moderately differentiated and 13 were diagnosed as poorly differentiated. We excluded the analysis of 6 cases of carcinoma in situ and 5 cases in which pathologists did not reach a consensus as to the degree of differentiation. Two groups divided by high or low expression were considered for statistical analysis. The most differentiated (grade I) ESCC samples showed a strong positive signal for galectin-7. Conversely, poorly differentiated (grade III) ESCC samples showed relatively low galectin-7 expression. A statistically significant association was observed between galectin-7 expression level and the differentiation of human ESCC (*P *= 0.045) (Table 2). Taken together, these results indicate that galectin-7 is up-regulated in ESCC and that its expression is related to the differentiation of ESCC.

**Table 2:** Comparison of ESCC differentiation degree with galectin-7 expression

Expression level	Differentiation degree			*P *value
		
	well	moderately	poorly	
low	4	5	10	0.045
high	8	9	3	

High expression levels correspond to tumors with either intense homogeneous or intense inhomogeneous Gal-7-dependent staining signals. Low expression levels correspond to tumors with either weak homogeneous staining signals or no detectable Gal-7 signal.

## Discussion

This study was designed to isolate and identify ESCC biomarkers using proteomic tools. Using 2-DE and mass spectrometry, we have successfully identified 27 proteins with different expression patterns in ESCC tissues. In previous ESCC proteomics studies, several differentially expressed proteins such as transgelin and tropomyosin were identified [14]. Among these 27 proteins, there are also several proteins we are interested, and may be further investigated, such as galectin-7, PEBP1, profilin1, TCTP and triosephosphate isomerase. Among them, galectin-7 was found to be rarely reported differentially expression in ESCC before. Besides, some reports showed that galectin-7 expression altered during the tumour progression in some kinds of carcinomas [27,29,40]. So, among these differentially expressed proteins, galectin-7 was selected for further verification and investigation.

There are several reports of galectin-7 expression in human cancers [30,41]. However, these reports suggest conflicting roles of galectin-7 in cancer progression [14]; galectin-7 appears to have dual roles, with anti- and pro-malignant features, and different expression levels in different cancers. Galectin-7 expression can be induced by p53 transfection in a human colon carcinoma cell line. Therefore, galectin-7 has been designated p53-induced gene 1 (PIG1) [42]. The anti-tumor roles of galectin-7 are associated with induction of apoptosis [43] or inhibition of cell proliferation [44]. In contrast, other reports have demonstrated that galectin-7 is up-regulated in other cancers such as hypopharyngeal squamous cell carcinoma [27]. One mechanism of the pro-tumor role of galectin-7 may involve induction of MMP-9, which plays an important role in cancer progression and metastasis [45]. Although proteomics has been used previously to characterize the molecular background of ESCC, this is the first report of an up-regulation of galectin-7 in ESCC tissues. Furthermore, the up-regulation was confirmed by western blotting and immunohistochemistry of clinical sample sets. Due to the epithelial of esophagus is one of restricted regions expressing galectin-7 [23], it is possible that the different expression pattern has special role in the progression of ESCC. Collectively, these results indicate that galectin-7 is a potential biomarker for ESCC.

We found that the level of galectin-7 expression was related to the degree of ESCC differentiation. The well-differentiated (grade I) ESCC samples showed a stronger positive signal for galectin-7 compared with poorly differentiated (grade III) ESCC samples. Because galectin-7 acts downstream of the p53 anti-tumor gene, up-regulation of galectin-7 expression in the early stages of ESCC progression may be a mechanism of organ self-protection. Consistent with our findings, other reports have indicated that galectin-7 has different expression pattern in the different degree of tumor differentiation in bladder squamous cell carcinoma [46] and glyctin-7 has both anti- and pro-malignant characteristics at different stages in the progression of thyroid cancer [40]. Because one of the main reasons for the high mortality rate of ESCC is that it is not detected until it has invaded surrounding organs and is at an advanced stage, the special up-regulation of galectin-7 in ESCC may be useful as a diagnosis marker of ESCC.

In this study, we found that galectin-7 was primarily localized in the nuclei of NEET cells, whereas it was distributed throughout the cytoplasm, nuclei and membranes of ESCC cells. The intracellular localization of certain proteins may be associated with tumor progression. For example, nuclear localization of galectin-7 was associated with the initiation of tumorigenesis in laryngeal squamous cell carcinomas [27]. Additionally, galectin-3 was localized to the nuclei of ESCC cells [47]. These results suggest that tumor progression of ESCC may be associated with a translocation of galectin-7 from the nucleus to the cytoplasm.

## Conclusions

Using proteomic analysis, we have identified galectin-7 as a potential biomarker of ESCC. Furthermore, we have confirmed the alteration in the expression of galectin-7 using a tissue microarray. These results strongly suggest that galectin-7 is involved in the development of ESCC and could potentially be served as a marker for ESCC.

## Competing interests

The authors declare that they have no competing interests.

## Authors' contributions

ZX and MXF initiated the study, participated in its design and coordination, carried out the study, performed the statistical analysis, and drafted the manuscript. FKZ and LJT were responsible for the experimental design and financial support. MD and MLY participated in sample preparation, trypsin digestion and MS identification. All authors read and approved the final manuscript.

## Pre-publication history

The pre-publication history for this paper can be accessed here:

http://www.biomedcentral.com/1471-2407/10/290/prepub

## Supplementary Material

Additional file 1 **Identification of proteins differentially expressed in esophageal cancer specimens by MALDI-TOF MS and MS/MS**. The figure is in the portable document format (Identification.pdf). Mascot MS scores were taken from the MS spectra search results using GPS Explorer software (Version 3.5). In this program, a Mascot score > 45 was considered significant. Click here for file

Additional file 2 **Expression patterns of differentially expressed proteins in esophageal cancer specimens and their biological functions**. The table is in the excel spreadsheet format (function.xls). (a) Ratio of protein expression level is the statistical analysis of the results from seven batches of 2D gels using ImageMasterTM software. (b) Biological function is based on the information from KEGG and ExPasy database. Click here for file

Additional file 3 **Functional classification of proteins**. The figure is in the portable document format (classification.pdf). Functional classification of differentially expressed proteins based on information from KEGG and ExPasy. Click here for file

Additional file 4 **Western blotting result of galectin-7 expression in several pairs of ESCC and NEET samples**. The figure is in the JPEG format (westernblot.jpg). Six representative pairs of tissue samples were resolved on 10% polyacrylamide gels and immunoblotted with an anti-galectin-7 antibody and a β-actin antibody as a loading control. T, tumour; N, normal. Click here for file

Additional file 5 **Sub-cellular localization of galectin-7 in four ESCC cell lines**. The figure is in the JPEG format (immunofluorescence.jpg). The sub-cellular localization and expression of Galectin-7 were examined by immunofluorescence staining in four ESCC cell lines, including KYSE 30, KYSE 70, KYSE 410 and TE-1. Cells were cultured on chamber slides, fixed with 4% paraformaldehyde in PBS, permeabilized using 0.2% Triton X-100, blocked using 3%BSA, and stained with anti-galectin-7(R&D Systems, Minneapolis, MN) as the primary antibody and DyLightTM549 conjugated anti-goat IgG(Thermo Fisher Scientific, Inc) as the second antibody. DAPI staining was used as inner control. Fluorescence images were collected and analyzed by laser scanning confocal microscopy (Nikon ECLIPSE TE2000-E). Expression of galectin-7 protein was detected ubiquitously in the cytoplasm, nuclei and membranes in four ESCC cell lines, which were consistent with the results of immunohistochemistry assay. Click here for file
