# Supplementary material for: Identification of galectin-7 as a potential biomarker for esophageal squamous cell carcinoma by proteomic analysis
Source: BMC Cancer. 2010 Jun 15;10:290. doi: 10.1186/1471-2407-10-290 (PMC3087317; doi:10.1186/1471-2407-10-290)
Supplement: Additional file 1 — Identification of proteins differentially expressed in esophageal cancer specimens by MALDI-TOF MS and MS/MS. The figure is in the portable document format (Identification.pdf). Mascot MS scores were taken from the MS spectra search results using GPS Explorer software (Version 3.5). In this program, a Mascot score > 45 was considered significant. [file 1471-2407-10-290-S1.PDF]

Additional file 1. Identification of proteins differentially expressed in esophageal cancer specimens by MALDI-TOF MS and MS/MS

Protein name	Accession No.	Gene name	Theoretical MW(Da)/pI	Mascot score	Matched peptide	Coverage (%)	Sequence confirmed by MS/MS	MS/MS score
Annexin A1	P04083	ANXA1	38690/6.57	334	16	42	^{114}TPAQFDADELR^{124}<br>^{215}GTDVNVFNTILTTR^{228}<br>^{189}SEDFGVNEDLADSDAR^{204}	72<br>29<br>39
Serpin B5 precursor	P36952	SERPINB5	42111.5/5.72	230	14	42	^{346}DELNADHPFIYIIR^{359}<br>^{48}GDTANEIGQVLHFENVK^{64}<br>^{322}VCLEITEDGGDSIEVPGAR^{340}	23<br>53<br>20
Transthyretin [Precursor]	P02766	TTR	15877/5.52	47	5	11	-	-
Thioredoxin	P10599	TXN	11729.7/4.82	106	8	21	^{73}CMPTFQFFK^{81}	32
Apolipoprotein A-I [Precursor]	P02647	APOA1	30758.9/5.56	243	12	32	^{121}VQPYLDDFQK^{130}<br>^{185}THLAPYSDELR^{195}<br>^{52}DYVSQFEGSALGK^{64}	22<br>60<br>21
Triosephosphate isomerase	P60174	TPI1	26652.7/6.45	103	7	24	^{34}VPADTEVVCAPPTAYIDFAR^{53}	22
Serpin B3	P29508	SERPINB3	44536.5/6.35	158	14	29	^{126}FYQTSVESVDFANAPEESR^{144}	83
Translationally controlled tumor protein	P13693	TPT1	19582.6/4.75	58	6	13	-	-

HHPeptidyl-prolyl cis-trans isomerase A	P62937	PPIA	18000.9/7.68	345	17	73	^{56}IIPGFMCQGGDFTR^{69}<br>^{77}SIYGEKFEDENFILK^{91}<br>^{2}VNPTVFFDIAVDGEPLGR^{19}	25<br>69<br>106
Profilin-1	P07737	PFN1	15044.6/8.44	148	8	47	^{57}SSFYVNGLTLGGQK^{70}	51
Cystatin-B	P04080	CSTB	11132.6/6.96	135	6	45	^{57}VHVGDEDFVHLR^{68}	62
Galectin-7	P47929	LGALS7	15065.8/7.03	317	16	85	^{76}GPGVPFQR^{83}<br>^{100}AVVGDAQYHHFR^{111}<br>^{33}FHVNLLCGEEQGSDAALHFNPR^{54}	34<br>54<br>54
Tropomyosin beta chain	P07951	TPM2	32830.6/4.66	325	21	37	^{92}IQLVEEELDR^{101}<br>^{168}KLVILEGELER^{178}<br>^{38}QLEEEQQALQK^{48}	65<br>53<br>29
Tropomyosin alpha-3 chain	P06753	TPM3	32798.7/4.68	301	19	41	^{168}KLVIIEGDLER^{178}<br>^{252}TIDDLEDELYAQK^{264}<br>^{92}IQLVEEELDRAQER^{105}	30<br>66<br>24
Myosin light chain 1	P05976	MYL1	21131.8/4.97	132	8	34	^{70}ITLSQVGDVLR^{80}<br>^{125}DQATYEDFVEGLR^{137}	28<br>32
Myosin light chain 3	P08590	MYL3	21917/5.03	292	13	55	^{82}ALGQNPTQAEVLR^{94}<br>^{126}DTGTYEDFVEGLR^{138}	54<br>80
Serum amyloid P-component [Precursor]	P02743	APCS	25371.1/6.1	74	7	21	-	-
Myosin light chain 6B	P14649	MYL6B	22749.7/5.56	79	6	23	^{139}GQGTYEDYLEGFR^{151}	36
Myosin regulatory light chain 2	P10916	MYL2	18777.4/4.92	252	17	76	^{41}DGFIDKNDLR^{50}<br>^{31}EAFTIMDQNR^{40}<br>^{92}GADPEETILNAFK^{104}	47<br>23<br>40
Myoglobin	P02144	MB	17172.9/7.14	56	5	27	-	-

Alpha-crystallin B chain	P02511	CRYAB	20146.4/7.7	284	11	43	^{12}RPFFPFHSPSR^{22}<br>^{57}APSWFDTGLSEMR^{69}<br>^{124}IPADVDPLTITSSLSSDGVLTVNGPR^{149}	45<br>25<br>81
Transgelin	Q01995	TAGLN	22596.4/8.87	90	7	24	^{21}KYDEELEER^{29}	30
Phosphatidylethanolamine-binding protein1	P30086	PEBP1	21043.7/7.01	94	7	42	^{63}LYTLVLTDPDAPSR^{76}	23
Carbonic anhydrase 1	P00915	CA1	28852.4/6.59	117	10	39	-	-
Carbonic anhydrase 3	P07451	CA3	29538.7/6.86	118	10	29	^{178}FDPSCLFPACR^{188}	38
Creatine kinase M-type	P06732	CKM	43073.9/6.77	150	16	35	^{117}GGDDLDPNYVLSSR^{130}<br>^{321}GTGGVDTAAVGSVFDVSNADR^{341}	24<br>37
Fructose-bisphosphate aldolase A	P04075	ALDOA	39395.3/8.3	114	13	39	-	-
