# Supplementary material for: Identification of galectin-7 as a potential biomarker for esophageal squamous cell carcinoma by proteomic analysis
Source: BMC Cancer. 2010 Jun 15;10:290. doi: 10.1186/1471-2407-10-290 (PMC3087317; doi:10.1186/1471-2407-10-290)
Supplement: Additional file 3 — Functional classification of proteins. The figure is in the portable document format (classification.pdf). Functional classification of differentially expressed proteins based on information from KEGG and ExPasy. [file 1471-2407-10-290-S3.PDF]

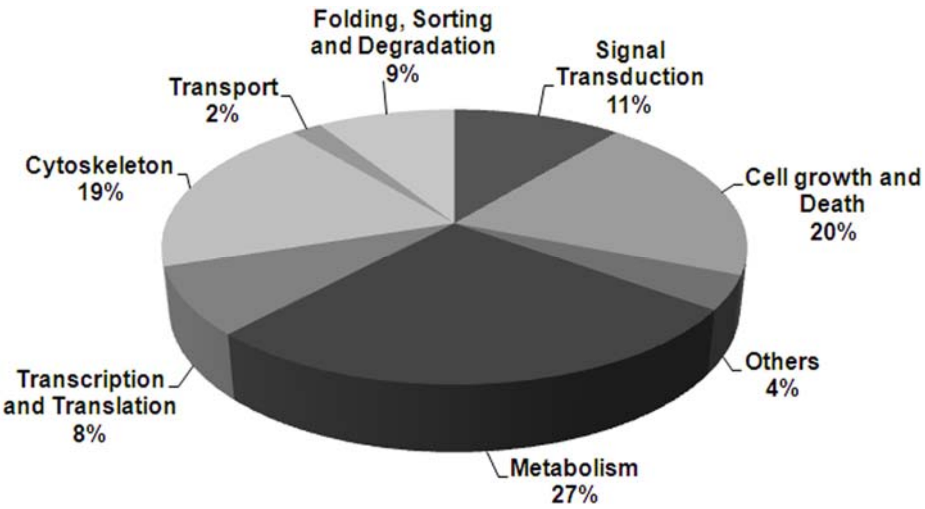

Additional file 3 - Functional classification of differentially expressed proteins based on information from KEGG and ExPasy.
