# Supplementary figures and images for: Identification of galectin-7 as a potential biomarker for esophageal squamous cell carcinoma by proteomic analysis
Source: BMC Cancer. 2010 Jun 15;10:290. doi: 10.1186/1471-2407-10-290 (PMC3087317; doi:10.1186/1471-2407-10-290)

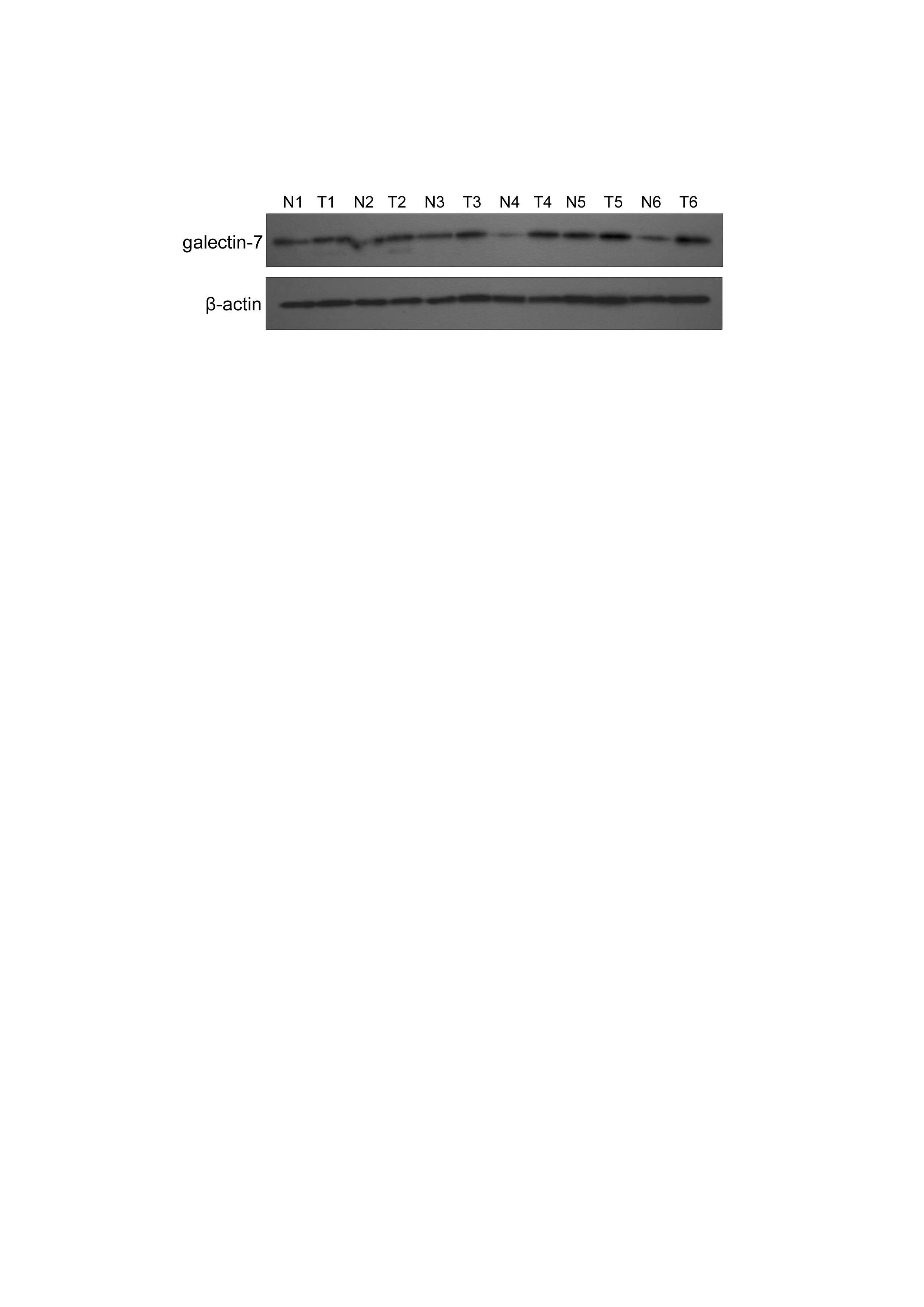

Supplement: Additional file 4 — Western blotting result of galectin-7 expression in several pairs of ESCC and NEET samples. The figure is in the JPEG format (westernblot.jpg). Six representative pairs of tissue samples were resolved on 10% polyacrylamide gels and immunoblotted with an anti-galectin-7 antibody and a β-actin antibody as a loading control. T, tumour; N, normal. [file 1471-2407-10-290-S4.JPEG]

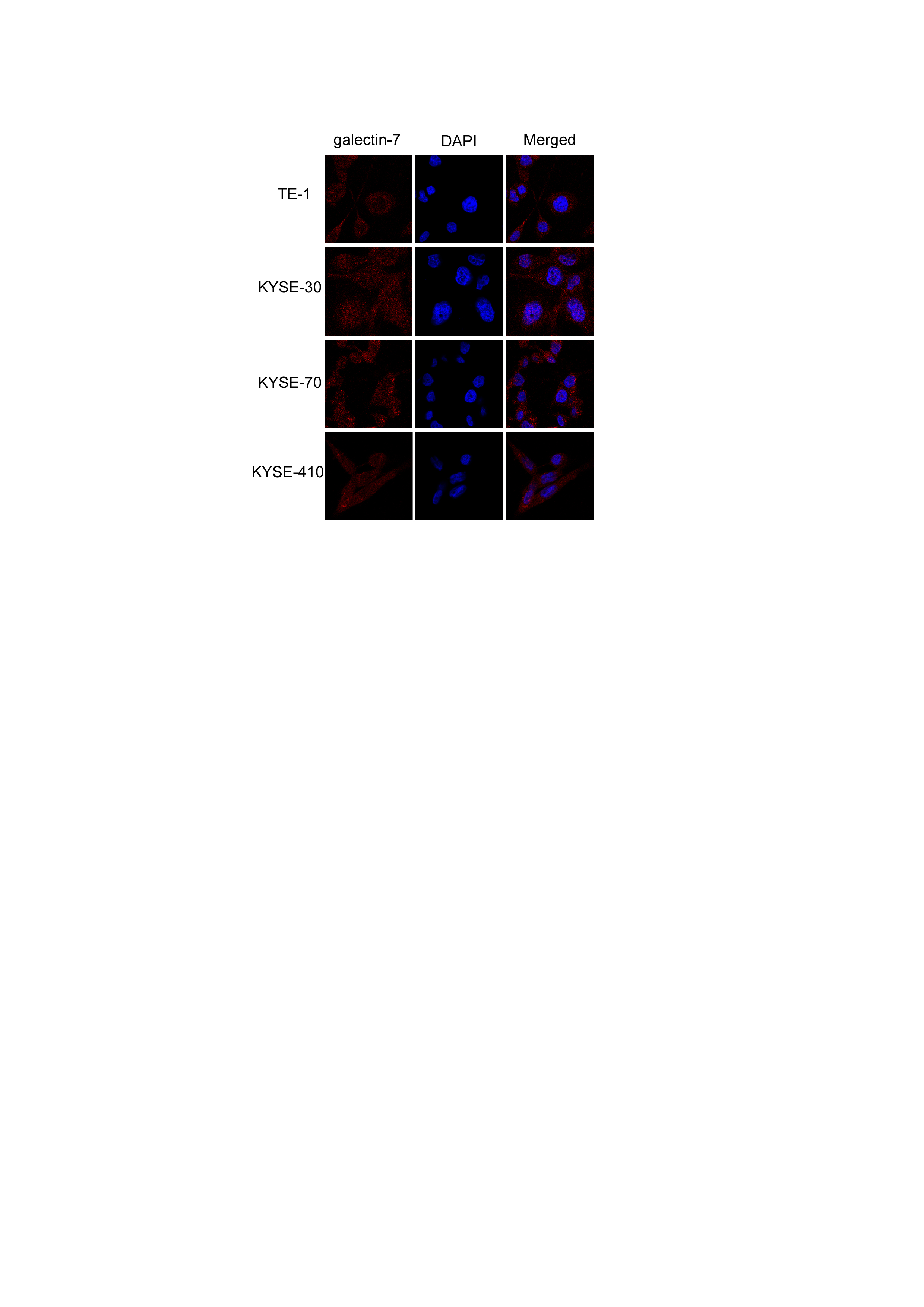

Supplement: Additional file 5 — Sub-cellular localization of galectin-7 in four ESCC cell lines. The figure is in the JPEG format (immunofluorescence.jpg). The sub-cellular localization and expression of Galectin-7 were examined by immunofluorescence staining in four ESCC cell lines, including KYSE 30, KYSE 70, KYSE 410 and TE-1. Cells were cultured on chamber slides, fixed with 4% paraformaldehyde in PBS, permeabilized using 0.2% Triton X-100, blocked using 3%BSA, and stained with anti-galectin-7(R&D Systems, Minneapolis, MN) as the primary antibody and DyLightTM549 conjugated anti-goat IgG(Thermo Fisher Scientific, Inc) as the second antibody. DAPI staining was used as inner control. Fluorescence images were collected and analyzed by laser scanning confocal microscopy (Nikon ECLIPSE TE2000-E). Expression of galectin-7 protein was detected ubiquitously in the cytoplasm, nuclei and membranes in four ESCC cell lines, which were consistent with the results of immunohistochemistry assay. [file 1471-2407-10-290-S5.JPEG]
